# Heritability and Genomic Architecture of Episodic Exercise-Induced Collapse in Border Collies

**DOI:** 10.3390/genes12121927

**Published:** 2021-11-29

**Authors:** Elaine M. Norton, Katie M. Minor, Susan M. Taylor, Molly E. McCue, James R. Mickelson

**Affiliations:** 1 Department of Veterinary Population Medicine, University of Minnesota, St. Paul, MN 55108, USA; norto253@umn.edu (E.M.N.); mccu0173@umn.edu (M.E.M.); 2 Department of Veterinary Biomedical Science, University of Minnesota, St. Paul, MN 55108, USA; minork@umn.edu; 3 Department of Small Animal Clinical Sciences, Western College of Veterinary Medicine, Saskatoon, SK S7N 5B4, Canada; sue.taylor@usask.ca

**Keywords:** episodic collapse, ataxia, border collie collapse, SNP array genotype data, genome-wide association analysis, bayesian, mixed linear model

## Abstract

An episodic nervous system disorder triggered by strenuous exercise, termed border collie collapse (BCC), exists in border collies and related breeds. The genetic basis of BCC is unknown but is believed to be a complex genetic disorder. Our goal was to estimate the heritability (h^2^_SNP_) of BCC, define its underlying genetic architecture, and identify associated genomic loci using dense whole-genome single-nucleotide polymorphism (SNP) genotyping data. Genotype data were obtained for ~440,000 SNPs from 343 border collies (168 BCC cases and 175 controls). h^2^_SNP_ was calculated to be 49–61% depending on the estimated BCC prevalence. A total of 2407 SNPs across the genome accounted for nearly all the h2_SNP_ of BCC, with an estimated 2003 SNPs of small effect, 349 SNPs of moderate effect, and 56 SNPs of large effect. Genome-wide association analyses identified significantly associated loci on chromosomes 1, 6, 11, 20, and 28, which accounted for ~5% of the total BCC h^2^_SNP_**.** We conclude that BCC is a moderately- to highly-heritable complex polygenetic disease resulting from contributions from hundreds to thousands of genetic variants with variable effect sizes. Understanding how much the BCC phenotype is determined by genetics and whether major gene mutations are likely to exist inform veterinarians and working/stock dog communities of the true nature of this condition.

## 1. Introduction

An episodic nervous system disorder triggered by strenuous exercise, termed border collie collapse (BCC), exists in border collies and border collie mixes. BCC is recognized worldwide in dogs used for working livestock, participating in agility and fly-ball competitions, or repeatedly retrieving a ball. Individuals susceptible to BCC can show clinical signs after 5 to 15 min of strenuous activity, particularly in warm weather [1,2,3,4]. The age range of onset of BCC is four months to seven years, with a median of ~two years). Typical collapse episodes include: disorientation; dull mentation or loss of focus; ataxia, swaying, staggering, and falling to the side; exaggerated lifting of each limb while walking and a choppy gait; increased extensor muscle tone; delayed limb protraction, dragging of the rear and/or forelegs, and crossing of the legs when turning (ataxia). It is common for the forelimbs and hindlimbs to be affected simultaneously, and for mentation to be altered during the episode. Gait and mentation both return to normal within 30 min of rest. Excessive panting and severe hyperthermia during episodes may be erroneously reported as heat stroke or heat intolerance, but affected dogs have no clinical or laboratory abnormalities consistent with such a diagnosis, and normal dogs participating in the sustained strenuous activity are comparably hyperthermic [5,6,7]. Episodic collapse, identical or highly similar to BCC, also exists in related breeds, including Australian shepherds, kelpies, bearded collies, Shetland sheepdogs, and whippets [8].

Most of our medical, epidemiological, and physiological understanding of BCC comes from studies conducted by Dr Sue Taylor and colleagues, who performed pre- and post-exercise evaluations of normal and affected border collies via standardized sheepherding and ball-chasing protocols [5]. Their work demonstrated that dogs with BCC have normal-appearing muscle biopsies, normal blood glucose, blood lactate, and serum electrolytes, and serum creatine kinase levels [5]. Collapsing border collies from the sheepherding and ball-chasing cohorts had similar clinical signs and laboratory findings, suggesting the same underlying basis for a collapse in both populations. Further, the “extreme hyperthermia” identified after exercise in dogs with BCC was also seen in normal exercise tolerant dogs performing the same exercise [7]. Lastly, the *DNM1* gene mutation responsible for a phenotypically dissimilar form of an exercise-induced collapse in Labrador retrievers and related breeds [9,10] was not identified in a large population of border collies meeting the diagnostic criteria for BCC [5].

A complete description of all genetic contributions to a trait, including all variants that influence it, the magnitude of each of their effects, their population allele frequencies, and their interactions with each other and the environment, is termed genetic architecture [11,12]. Narrow-sense heritability (h^2^) refers specifically to the proportion of risk attributable to the additive genetic variance and can be estimated using SNP genotype data (h^2^_SNP_). Knowledge of the genetic architecture is essential for efficient and successful genetic research on novel traits, particularly those unlikely to have a simple genetic basis. The objective of this study was to estimate the h^2^_SNP_ of BCC and define its underlying genetic architecture and associated genomic loci through computational analyses of dense whole-genome SNP genotyping data.

## 2. Materials and Methods

### 2.1. Sample Collection and Criteria

A total of 343 border collies, 168 BCC cases, and 175 controls, were included in this study. Among these dogs, 246 were from the US, 65 were from Canada, 15 were from the United Kingdom, and the remainder were from 12 other countries. Samples were collected at herding trials or through outreach via our website [13]. Of the 168 cases, 85 were female (28 intact and 57 spayed) and 83 were male (27 intact and 56 castrated). The mean age at the onset of clinical signs for the cases was 1.9 years (range: 4 months to 5 years) with an average age at the time of sampling of 4.9 years (range: 9 months to 15 years). For the 175 controls, 93 were female (36 intact and 57 spayed) and 82 were males (40 intact and 41 castrated). The mean age at sampling for the controls was 8.1 years (range: 2 to 16 years). Follow-up information obtained from 101 control dogs (range post initial sample submission of 1–11 years, mean ~3 years) found that all had remained symptom-free. The 23 control dogs sampled at <5 years of age were confirmed to be controls by at least 5 years of age at this follow-up.

For each submission, clinical features were collected from owners via a 48-question survey [13]. Owners were also requested to upload a video of the dog during work for evaluation by an observer trained in the diagnosis of BCC. Criteria for inclusion for BCC cases were developed from our previous studies [4,5] and include: dogs with a history of at least two episodes of typical BCC collapse and video evidence that well-illustrated a collapse episode; age of the first episode was <5 years of age; all episodes occurred during or immediately after strenuous activity; episodes were more likely to occur with excitement and heat; episodes were sudden onset; mentation was altered, with a loss of focus and sometimes a loss of balance; all four legs were affected (rear may be more severe); affected legs were stiff, with increased extensor tone, delayed limb protraction and toe scuffing during walking; no systemic or autonomic signs; episodes usually last 5–15 min; normal between episodes; and episodes were intermittent.

Dogs were excluded from the study if their age of onset was greater than 5 years, the episodes were not always exercise-induced, the episodes were very brief (1–2 min), systemic or autonomic signs were evident, if the episodes were determined to be seizures, or if the dog exhibited clinical signs of pain during an episode. The rationale for exclusion was that these types of episodes of exercise intolerance could be attributed to other disorders such as joint pain, heart failure, pulmonary hypertension, anemia, heart rhythm disturbances, laryngeal paralysis, lung disease, low blood sugar, low blood cortisol, cauda equina syndrome, myasthenia gravis, epilepsy, and muscle disease.

Dogs who routinely participated in strenuous activity without evidence of collapse or owner-reported heat intolerance, that were 5 years of age or older, were eligible to serve as controls. We considered the false-negative calling of a BCC dog as a control to be unlikely given our criteria of 5 years of age, along with participation in field trials. However, it was possible that BCC-susceptible dogs may only develop symptoms in the heat, and dogs residing in cool temperature climates may not exhibit clinical signs, leading to a false negative. This likelihood is small given that most dogs enrolled trained in all seasons, and many traveled for competitions and were exposed to a variety of climates.

### 2.2. Genotype Data Processing and Imputation

DNA was isolated from 3–5 mL whole blood or 1–3 cheek swabs using the appropriate protocols provided in the Gentra PureGene Handbook. The SNP genotyping platform used depended on the year of sampling and available arrays; 121 dogs were genotyped on the Illumina 170K SNP array (170,000 SNPs), 184 were genotyped on the Affymetrix 670K SNP array (670,000 SNPs), 35 were genotyped on the Affymetrix 1.2M SNP array (1.2 million SNPs), and three dogs had whole-genome sequencing available (PCR-free libraries, −680 bp inserts, Illumina HiSeq 4000 sequencer, ~125 million 2 × 150 bp paired-end reads, mapped against CanFam3.1). To create a uniform dataset, higher density (1.2M and WGS) SNP genotype data was masked down to the 670K array SNP content, and lower density (170K) SNP genotype data were imputed up to the 670K array content.

We first integrated the genotype data into our internal database of SNP genotyping data from all publicly available canine projects and those private to our group. This yielded a >4000 dog database representing more than 100 breeds which served as a multi-breed reference population for imputation of the border collie SNP data to the 670K density array with Beagle 4.1 [14]. The target and reference panels were pruned to remove SNPs with more than two alleles, minor allele frequency less than 0.001, and discordant genotypes within a multibreed panel of 360 dogs genotyped at both 170K and 670K.

Quality control on the data was performed in PLINK 1.9 [15] where SNPs were excluded for genotyping rates <90.0%, minor allele frequencies <5%, differential missingness between cases and controls (*p* < 0.01), or deviation from Hardy Weinberg Equilibrium in controls (*p* < 0.001). Samples were excluded for SNP genotyping rates <97% for blood and <93% for cheek swab samples (per Affymetrix). After the above quality control and pruning steps, ~440,000 SNPs remained in the analysis.

**Figure 1:**
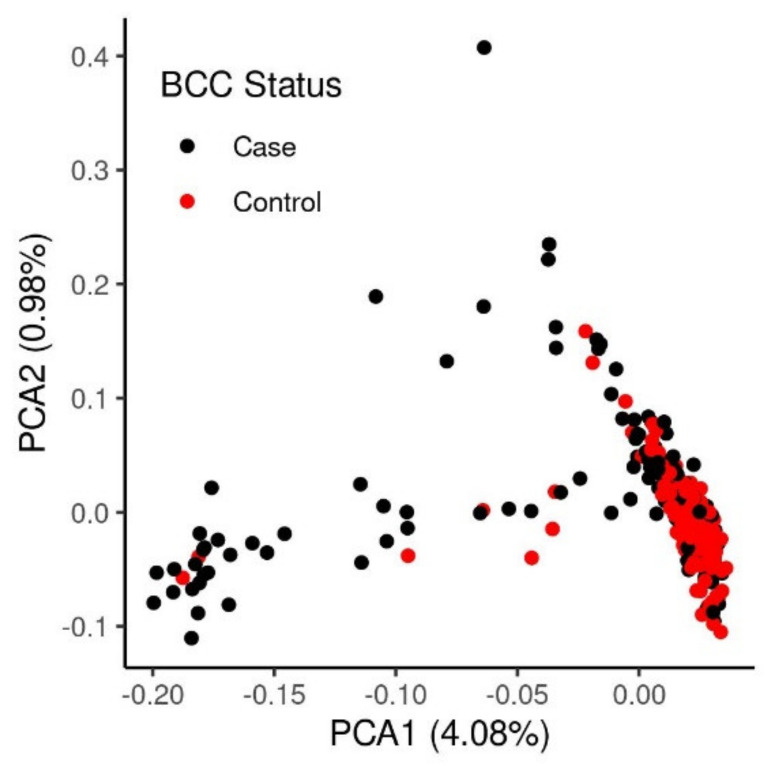
Principal Components Analysis of the border collie SNP array genotype data. Inset red dots refer to cases and grey dots refer to controls.

An identity-by-state (IBS) analysis using PLINK found that the mean IBS across groups was 0.75 (sd: 0.02), while the mean IBS amongst cases was 0.74 (sd: 0.02) and amongst controls was 0.76 (sd: 0.01). Pairwise comparisons indicated that cases were more similar to other cases and controls were more similar to other controls, although these differences were small. A plot of the first two principal components generated from the imputed SNP genotype data is presented in Figure 1. A degree of population stratification is not unexpected in animal studies and was accounted for by including a standard or weighted genetic relationship matrix within our analyses.

### 2.3. Model Analysis

Sex (intact female, spayed female, intact male, or castrated male), region (country), genotyping array (batch effect), and line (conformation versus working dog) were assessed as covariates with the best subsets logistic regression using log-likelihood and Pearson’s chi-square goodness of fit tests (Table S1). Based on these analyses, region, genotyping array, and lineage remained in the models as fixed effects. Due to the age criteria for inclusion in the study, age itself was not included in the model.

### 2.4. Estimation of Heritability

SNP-based heritability (h^2^_SNP_) was estimated from the ~440K imputed SNPs after excluding the X-chromosome using a mixed linear model implemented in the software program Genome-wide Complex Trait Analysis (GCTA) [16] with the inclusion of the fixed effects and a genetic relationship matrix (GRM). Briefly, the GCTA algorithm estimates SNP-based narrow-sense heritability (h^2^_SNP_) by simultaneously fitting all available SNPs into the model using a maximum likelihood statistic. To account for population substructure within our cohort, a weighted genetic relationship matrix (wGRM) was obtained from the software program Linkage Disequilibrium Adjusted Kinship (LDAK) [17], which adjusts for SNPs in linkage disequilibrium to prevent inflation of h^2^_SNP_ estimates. The prevalence of BCC in the general population is unknown but could be estimated from our previous work with breed associations and herding trial groups to be between 5–10%. To control for ascertainment bias h^2^_SNP_ estimates were repeated with a disease prevalence of 5%, 8%, and 10%.

### 2.5. Determination of Genomic Architecture

An estimation of genomic architecture was constructed from the imputed SNP data using the software program BayesR [18]. In this model, SNP effects are estimated simultaneously using Gibbs sampling to estimate the percentage of SNPs with a small to large effect size under the assumption that the true SNP effect falls into a four-component mixture of normal distributions (SNPs with zero, small, moderate, or large effect). To account for covariates, the residuals from the logistic regression model with case-control status as a binary outcome variable and country, genotyping array, and line (conformation versus working dog) as predictor variables were input as the phenotype of interest.

We utilized the modified BayesR code by Mollandin et al. [19] to estimate the proportion of genetic variation explained by the SNPs on the array. This code allows for the extrapolation of the posterior variance of estimated SNPs effects at each iteration.

### 2.6. Genome-Wide Association Analysis

We performed genome-wide association analyses (GWA) to identify regions of the genome associated with BCC. The Genetic Type 1 Error Calculator (GEC) [20] was used to calculate the suggestive (1.0 × 10^−5^) and genome-wide significance (2.3 × 10^−7^) thresholds based on the effective number of independent tests. A mixed model analysis in the software program Genome-Wide Efficient Mixed Model Analysis v 0.98.4 (GEMMA) [21] was used with the imputed genotype data and the inclusion of the fixed effects and a standard GRM. We also used an improved mixed linear model which we previously utilized to increase power in cohorts with a relatively small sample size [22]. Briefly, this algorithm implements a three steps process. The first step divides the genome into chromosomes and then bins SNPs by 500 kb regions. The software program Bayesian Sparse Linear Mixed Model (BSLMM) in GEMMA is then used to determine which SNP in each bin had the highest model frequency. This SNP, and the two adjacent SNPs, are then used in step 2 to create a select SNP GRM based on the likelihood ratio test for improvement of the null model. In step 3, a linear mixed model is performed on all SNPs using the software program FastLMM with the inclusion of the select SNP GRM. To avoid proximal contamination, the tested SNP, along with all SNPs within 1 Mb of the tested SNP, is removed from the select SNP GRM during analysis.

The boundaries of the regions associated with BCC identified on GWA were defined based on the breakdown of linkage disequilibrium (LD) as previously described [22]. Briefly, PLINK was used to measure pairwise LD for each SNP exceeding the suggestive or genome-wide significant threshold in the GWA [15]. A custom code was then utilized to determine when all SNPs dropped below an *r*^2^ of 0.3 for 100 kb both 5′ and 3′ of the widest peak. These LD-defined boundaries were then used to determine the total proportion of genetic variance explained by each identified GWA locus using the results from the modified BayesR code [19]. These regions were also used to determine the number of known positional candidate genes using the R Bioconductor package biomaRt [23] with the CanFam3.1 reference genome.

## 3. Results

### 3.1. Assessment of the Heritability and Genetic Architecture of BCC

The h^2^_SNP_ of BCC, with different estimates of prevalence in the population, was estimated using GCTA and the wGRM from LDAK. h^2^_SNP_ was 49% at a disease prevalence of 5% (SE = 15%), 57% at a disease prevalence of 8% (SE = 17%), and 61% at a disease prevalence of 10% (SE = 18%), indicating that BCC is a moderately- to highly- heritable trait (Table 1).

**Figure 2:**
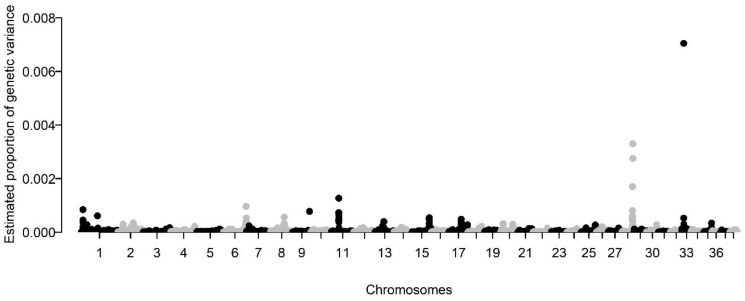
Posterior estimates of the per-SNP proportion of genetic variance explained across all ~440K SNPs. The 38 canine autosomes are plotted on the *x*-axis and the estimated proportion of the genetic variance explained is presented on the *y*-axis. Each dot represents a single SNP. each dot refers to a SNP and there is no significance to grey vs black dots. The colors simply are alternating chromosomes.

We next utilized BayesR to derive the genetic architecture of BCC after accounting for fixed effects. SNP-based heritability of BCC in our border collie population was explained by 2407 SNPs. Of these SNPs, 83.2% (*n* = 2003) were of small effect [10 × 10^−4^ × genetic variance], whereas 14.5% (*n* = 349 SNPs) were of moderate effect [10 × 10^−3^ × genetic variance], and 2.3% (*n* = 56 SNPs) were of high effect [10 × 10^−2^ × genetic variance]. The model estimated that these 2407 SNPs accounted for virtually all the h^2^_SNP_ of BCC, with the 2003 SNPs of small effect accounting for 21.0% of the genetic variation, the 349 SNPs of moderate effect accounting for 36.9% of the genetic variation, and the 56 SNPs of large effect accounting for 34.5% of the genetic variation. The proportion of genetic variation explained by the SNPs on the array was then assessed [18], where it was determined that the largest percentage of genetic variance (posterior variance of SNP effect >1.0 × 10^−3^) was explained from SNPs located on chromosomes 11, 28, and 33 with a small percentage of the genetic variance (posterior variance >5.0 × 10^−4^ and <1.0 × 10^−3^) explained by SNPs on chromosomes 1, 6, 8, 9, 13, 15, and 17 (Figure 2). The summation of these SNPs explained 41% of the total h^2^_SNP_.

### 3.2. Genome-Wide Association Analysis

**Figure 3:**
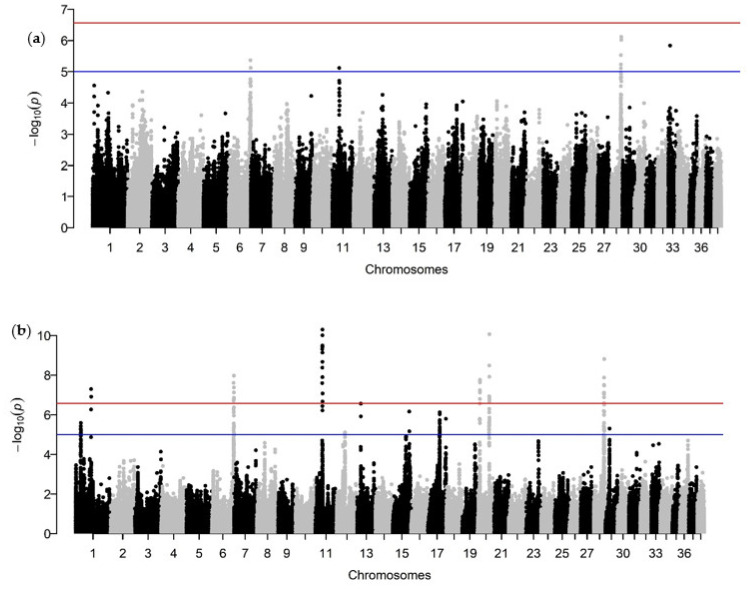
Manhattan plots of the genome-wide association results for BCC using GEMMA (**a**) and an improved mixed linear model (**b**). The 38 autosomes are plotted on the *x*-axis and the −log10 of the *p*-value for the comparisons of allele frequencies of each SNP between cases and controls is presented on the *y*-axis. Each dot represents a single SNP. The suggestive and genome-wide significant thresholds are represented by the blue and red lines, respectively. The genomic inflation factor (λ) was 0.98 for GEMMA and 0.90 for the improved linear mixed model.

The ~440K SNP genotype data was used with two different analysis methods to identify major chromosomal loci where genes for BCC lie. Using the mixed linear model implemented in GEMMA, regions on chromosomes 6, 11, 28, and 33 exceeded the suggestive threshold (Figure 3a). The improved linear mixed model also identified the same regions on chromosomes 6, 11, and 28, which exceeded our genome-wide significant thresholds, as well as additional loci on chromosomes 1 and 20 (Figure 3b). SNPs on chromosomes 12, 13, 15, and 17 exceeded the suggestive threshold for the improved linear mixed model. LD-based genomic coordinates, fine scale alignments, and the total proportion of genetic variance explained by each of these significant loci are provided in Table 2.

## 4. Discussion

We have utilized a moderately large cohort with an extensive SNP genotyping dataset to demonstrate that the heritability of BCC is between 49–61%, depending on disease prevalence, and hundreds of alleles contribute to BCC risk. We also identified multiple genomic loci associated with BCC. Together, these data suggest that BCC is a complex moderately- to highly heritable disease, where many genetic variants, coupled with environmental factors contribute to BCC risk. The results have practical implications for veterinarians, breeders, owners, and trainers, in addition to clarifying obstacles researchers will face in determining BCC’s molecular basis and developing tests to quantify an individual’s genetic risk in developing BCC.

Traditionally, studies that estimate complex trait heritability in dogs have utilized pedigree analyses; however, this approach may be overestimating heritability as a consequence of relatively small sample sizes and highly related individuals, leading to confounding from the shared environment and ascertainment bias. SNP genotyping array data makes the estimation of heritability highly feasible and allows for more precise estimates using a large group of unrelated individuals [11,12]. This approach also enables the planning of genomic mapping projects with reasonable expectations of identifying contributing variants and developing predictive tests, even before the discovery of functional alleles.

In order to obtain unbiased estimates of genetic variation and reduce spurious associations on genome-wide analyses, relevant confounders need to be included in the final model. Country and line (conformation vs. working) were included as fixed effects due to regional breeding preferences and distinct population stratification which has been previously reported between show and working dog lines [24]. Array was also included as a fixed effect as, prior to obtaining a uniform set of SNPs, individuals in our cohort were genotyped on one of five genotyping platforms over a span of several years. Batch effects both within and between arrays have been shown to introduce bias which could affect the results of genetic studies [25]. We also evaluated for a sex predilection in our cohort by including sex (intact female, spayed female, intact male, and castrated male) as a potential covariate, but it did not improve the model (Table S1). Comparison of the results from the h^2^_SNP_ estimates with and without the inclusion of fixed effects did not significantly alter the results (Table S2).

We opted to use a wGRM, which adjusts the GRM to prevent over- or underestimation of h^2^_SNP_ due to SNPs in linkage disequilibrium. When the results obtained with the wGRM were compared to those with the standard GRM we found that the estimates only differed by 2%, suggesting that linkage disequilibrium had a minimal impact on our estimates (Table S2). We also included an estimate of the population prevalence in order to reduce ascertainment bias inherent in case-control studies [26]. Since epidemiological studies for BCC are unavailable, we used a range of 5–10% prevalence, informed by our previous work. Using a prevalence of 5%, 8% and 10% resulted in h^2^_SNP_ values of 49%, 57%, and 61%, respectively. This range is a consequence of the large percentage of cases in our cohort and reflects the importance of transforming the estimates on the liability scale to prevent the overestimation of h^2^_SNP_. Nevertheless, these results confirm that genetics is contributing to a large percentage of the phenotypic variation of BCC.

Knowledge of the genetic architecture of a trait is an important step for determining appropriate genomic methods for identification of the specific causal variants, as well as understanding the number of variants having a moderate to high impact, as these have the most relevance for predicting an individual’s genetic risk and are more likely to result in targeted therapies. BayesR determines the genetic architecture of a trait by assigning priors to bins representing SNPs of null, low, moderate, or high effect. Gibbs sampling is then used to determine the posterior probability that an SNP is assigned to each bin, and the final model parameters are determined based on the posterior mean across iterations. We determined that virtually all of our conservative estimates of h^2^_SNP_ could be explained by 2407 SNPs across the genome. The majority of these SNPs (83.2%, *n* = 2003) were predicted to have a small effect whereas the remaining 405 SNPs were predicted to have a moderate or high effect, which is consistent with the genetic architecture of complex traits [27]. Thus, these results indicate that BCC is a complex, polygenic trait with thousands of variants across the genome contributing to the phenotype and hundreds of variants having a moderate to a large impact.

Simple dominant or recessive (single-gene Mendelian) traits, with heritabilities approaching 1.0 (100%), can frequently have their causative mutations identified by GWA studies to define the causative locus, combined with Sanger or whole-genome sequencing (WGS). Due to limitations in sample population sizes and structure, and in some cases SNP genotyping array densities, our ability to investigate canine complex/polygenic genetic diseases with moderate heritabilities is far more limited when using software programs optimized for human data (sample sizes in the thousands with reduced relatedness). Thus, it is not surprising that we were not powered to detect loci exceeding our genome-wide significant threshold with GEMMA. However, we have previously described an improved linear mixed model for the analysis of complex traits with small sample sizes [22]. This approach improved our power to detect the loci on chromosomes 6, 11, and 28, as well as identify additional loci on chromosomes 1 and 20. Loci on chromosomes 12, 13, 15, and 17 exceed the suggestive threshold on our improved linear mixed model, potentially contributing to BCC as well. An additional follow-up to verify these loci in an independent population and to identify the specific genetic risk alleles is required.

In BayesR, the objective is not to estimate individual SNP effects, but to determine the number of SNPs contributing to a phenotype based on a distribution of probabilities that an SNP falls into one of four effect sizes. However, a recent modification of the BayesR code allows for the extrapolation of the posterior variance of the per-SNP effects at each iteration, providing an estimate of the percentage of total genetic variance explained by each SNP. In our dataset, the largest percentage of genetic variance (posterior variance of SNP effect >1.0 × 10^−3^) was explained from SNPs located on chromosomes 11, 28, and 33 with a smaller percentage (posterior variance >5.0 × 10^−4^ and <1.0 × 10^−3^) explained by SNPs on chromosomes 1, 6, 8, 9, 13, 15, and 17. Combined, these loci explained 41% of the total h^2^_SNP_. Intriguingly, the majority of the SNPs identified on this analysis were also SNPs that exceeded the suggestive or genome-wide threshold in the GWA using the improved linear mixed model, accounting for approximately 5% of the total h^2^_SNP_. The SNP on chromosome 33 had the largest individual SNP posterior variance (~0.07%). This SNP also exceeded the suggestive threshold for our analysis on GEMMA; however, single SNP regions are often spurious associations and further analysis is required to determine its validity.

Notably, the modified BayesR model has been found to be sensitive to several conditions which could under or overestimate the per-SNP genetic variance, including: (1) the presence of the true quantitative trait loci (QTL) on the genotyping platform, (2) the effect size of the QTL, and (3) the number of SNPs in linkage disequilibrium with the QTL. Further caution must be taken when comparing analyses across different approaches, particularly Bayesian and frequentist statistics. Nevertheless, this provides compelling evidence for prioritizing further exploration of the LD-defined regions identified on chromosomes 6, 11, and 28 as these are likely harboring variants of high effect.

Episodic collapse, identical or highly similar to BCC, also exists in border collie mixes, and related breeds, including Australian shepherds, kelpies, bearded collies, Shetland sheepdogs, and whippets. SNP data have demonstrated the close genetic relationship of these breeds to border collies [28], supporting a shared genetic basis for their syndromes. As a preliminary analysis, we estimated h^2^_SNP_ in an Australian shepherd population of 41 cases and 125 controls. Our estimates were similar to those found in the border collies, albeit with large standard errors due to the small population size in this cohort (Table S3). Although this provides initial support for a genetic contribution to BCC in other breeds, it requires follow-up in a larger population to be confirmed.

Our descriptions of typical signalment, clinical features, laboratory criteria [1,2,3,4,5], and now the complex genetic basis of BCC, allows veterinarians and dog owners to recognize the condition and predict its significance in individual cases. A future goal could be the generation of sufficient whole-genome sequencing and SNP prioritization pipelines, coupled with increased sample population sizes, to enable design and validation of a diagnostic genotyping assay and genetic risk model to predict an individual’s risk for BCC, enable appropriate scaled interventions, and planned breeding strategies.

## 5. Conclusions

BCC is a common condition affecting the health and well-being of many related herding breeds that work stock, participate in agility and fly-ball competitions, or repeatedly retrieve a ball. Our analyses determined that the heritability of BCC in border collies is in the range of 49–61% and that hundreds of alleles of moderate to large effect contribute to BCC risk. This makes BCC a moderately- to a highly heritable complex genetic disorder, where many genetic variants, in combination with environmental factors, contribute to the likelihood of developing the condition.

## Figures and Tables

**Table 1:** SNP-based heritability (h^2^_SNP_) estimates of BCC in border collies obtained at three different estimates of disease prevalence. SNP-based heritability was determined with the inclusion of a weighted standard genetic relationship matrix (wGRM) and the fixed effects of region, SNP array, and line (conformation versus working dog).

Prevalence	h^2^_SNP_	SE	*p*-Value
0.05	0.49	0.15	3.19 × 10^−5^
0.08	0.57	0.17	3.19 × 10^−5^
0.10	0.61	0.18	3.19 × 10^−5^

**Table 2:** BCC-associated genomic loci identified with the improved linear mixed model. The total number of SNPs exceeding the suggestive threshold (Total SNPs) and genome-wide significant threshold (Significant SNPs) is provided. Genomic coordinates are based on the LD-defined regions with the minimum (Min LD Region) and maximum (Max LD Region) base pair positions provided. Total percent of h^2^_SNP_ explained (Total % h^2^_SNP_) is based on the summation of the per-SNP posterior estimates of genetic variance for each GWA region.

CHR	Total SNPs	Significant SNPs	Min LD Region	Max LD Region	Total % h^2^_SNP_	Positional Coding Genes	OtherPositional Genes
1	10	1	17039965	20022599	0.36%	13	13
	3	2	54214259	54884985	0.15%	7	2
6	21	8	75980913	77466517	0.66%	5	10
11	15	13	23858342	25374425	0.64%	8	6
12	6	0	28213995	29672357	0.03%	3	3
13	2	0	11870489	12736069	0.01%	2	0
15	2	0	57796133	58553452	0.05%	3	1
17	17	0	39495267	42135498	0.45%	16	10
20	10	6	6702324	8206842	0.28%	13	2
	26	10	39976489	41516852	0.20%	45	7
28	3	2	34428475	34905524	0.05%	2	5
	23	8	35367805	37688937	1.85%	9	10

## Data Availability

The files are uploaded to Open Science Framework and will be made publicly available upon acceptance of the article (https://osf.io/auv96/?view_only=31cc286833fe4fba90a916c6b63f3824, accessed on 7 November 2021).

## Supplementary Materials

The following are available online at https://www.mdpi.com/article/10.3390/genes12121927/s1, Table S1: Assessment of covariates for genomic analyses, Table S2: Comparison of SNP-based heritability (h^2^_SNP_) estimates using two different genetic relationship matrices and three different disease prevalence, and Table S3: SNP-based heritability (h^2^_SNP_) estimates for BCC in a cohort of Australian shepherds.
