# Supplementary material for: Heritability and Genomic Architecture of Episodic Exercise-Induced Collapse in Border Collies
Source: Genes (Basel). 2021 Nov 29;12(12):1927. doi: 10.3390/genes12121927 (PMC8701027; doi:10.3390/genes12121927)
Supplement: Supplementary file 1 [file genes-12-01927-s001.zip › genes-1479468-supplementary.pdf]

genes MDPI

Table S1. Assessment of covariates for genomic analyses. The best subsets for logistic regression from log likelihood and Pearson's chi-square goodness of fit tests for covariate analysis are provided. Sex (intact female, spayed female, intact male, and castrated male), region (country), genotyping array (batch effect), and line (conformation versus working dog) were assessed as potential fixed effects. Abbreviations: DF, degrees of freedom.

Covariates	Log Likelihood	DF	P-Value
None	-237.7	1	-
Sex	-237.6	4	6.37e-01
Array	-230.3	6	1.20e-02
Sex + Array	-230.3	9	2.21e-02
Region	-213.8	15	1.43e-05
Sex + Region	-213.1	18	1.61e-05
Array + Region	-207.2	20	2.80e-06
Sex+Array+Region	-206.6	23	3.20e-06
Line	-228.7	2	2.26e-05
Line+Sex	-228.5	5	1.02e-04
Line+Array	-221.2	7	1.11e-05
Line+Region	-205.4	16	4.08e-08
Line+Sex+Array	-221.1	11	2.49E-05
Line+Sex+Region	-204.6	20	4.79e-08
Line+Array+Region	-198.7	21	1.50e-08
Line+Sex+Array+Region	-199.40	24	172e-08

Table S2. Comparison of SNP-based heritability ($h2_{SNP}$) estimates using two different genetic relationship matrices and three different disease prevelances. h^2_{SNP} was estimated with the inclusion of the standard genetic relationship matrix (GRM), and a weighted genetic relationship matrix (wGRM). Estimates were performed including no fixed effects and the fixed effects of region, SNP array, and line (conformation versus working dog).

		No Fixed Effects			Inclusion of Fixed Effects		
**GRM**	**Prev**	**$h2_{SNP}$**	**SE**	**P-Value**	**$h2_{SNP}$**	**SE**	**P-Value**
GRM	0.05	0.47	0.13	4.30e-10	0.48	0.15	3.66e-05
	0.08	0.55	0.15	4.30e-10	0.56	0.17	3.66e-05
	0.10	0.59	0.16	4.30e-10	0.59	0.18	3.66e-05
wGRM	0.05	0.49	0.13	3.47e-10	0.49	0.15	3.19e-05
	0.08	0.57	0.15	3.47e-10	0.57	0.17	3.19e-05
	0.10	0.61	0.16	3.47e-10	0.61	0.18	3.19e-05

The Table shows that at any given estimate of BCC population prevalence (0.05, 0.08, and 0.10) the h^2_{SNP} estimate determined with the GRM was just slightly less than the estimate determined with the wGRM. These data also shows that addition of region (county of origin), SNP array platform (batch effect) and line (conformation versus working dogs) as covariates had only a minimal influence on the h^2_{SNP} estimates determined using the GRM and the wGRM.

Table S3. SNP-based heritability ($h2_{SNP}$) estimates for BCC in a cohort of Australian shepherds. 167 Australian shepherds (41 cases and 126 controls) were collected, genotyped on SNP arrays, and the data imputed and pruned as described in Materials and Methods. Heritability estimates were performed both with the standard and weighted genetic relationship matrix (wGRM). Array and region were included as fixed effects determined by best subsets for logistic regression from log likelihood and Pearson's chi-square goodness of fit tests (data not shown).

GRM	Prev	$h2_{SNP}$	SE	P-Value
GRM	0.05	0.66	0.43	4.15e-02
	0.08	0.76	0.50	4.15e-02
	0.10	0.81	0.53	4.15e-02
wGRM	0.05	0.69	0.44	4.62e-02
	0.08	0.79	0.51	4.62e-02
	0.10	0.85	0.55	4.62e-02
